# Plasma cell infiltration and treatment effect in breast cancer patients treated with neoadjuvant chemotherapy

**DOI:** 10.1186/s13058-021-01477-w

**Published:** 2021-10-29

**Authors:** Asumi Sakaguchi, Yoshiya Horimoto, Hiroko Onagi, Daiki Ikarashi, Takayuki Nakayama, Tetsuya Nakatsura, Hideo Shimizu, Kuniaki Kojima, Takashi Yao, Toshiharu Matsumoto, Kanako Ogura, Shigehisa Kitano

**Affiliations:** 1 Department of Diagnostic Pathology, Juntendo University Nerima Hospital, 3-1-10 Takanodai, Nerima-ku, Tokyo, 177-8521 Japan; 2 Department of Human Pathology, Juntendo University School of Medicine, Tokyo, Japan; 3 Department of Breast Oncology, Juntendo University School of Medicine, 2-1-1 Hongo, Bunkyo-ku, Tokyo, 113-0033 Japan; 4 Division of Cancer Immunotherapy, Exploratory Oncology Research and Clinical Trial Center, National Cancer Center Hospital, 6-5-1 Kashiwanoha, Kashiwa, Chiba 277-8577 Japan; 5 Department of General Surgery, Juntendo University Nerima Hospital, 3-1-10 Takanodai, Nerima-ku, Tokyo, 177-8521 Japan; 6 Division of Cancer Immunotherapy Development, Advanced Medical Development Center, The Cancer Institute Hospital of Japanese Foundation for Cancer Research, 3-8-31 Ariake, Koto-ku, Tokyo, 135-8550 Japan

**Keywords:** Breast cancer, Plasma cell, Tumour-infiltrating lymphocyte, Neoadjuvant chemotherapy, Local immune microenvironment, Multiplexed fluorescent immunohistochemistry

## Abstract

**Background:**

Tumour-infiltrating lymphocyte (TIL)-high breast tumours have a high rate of pathological complete response (pCR) with neoadjuvant chemotherapy. In our routine pathological diagnoses of biopsy specimens from pCR cases, we have observed a high infiltration of plasma cells (PCs). A positive correlation of PCs with favourable patient outcome has recently been reported, but little is known about how PCs contribute to local tumour immunity.

**Methods:**

We retrospectively examined biopsy specimens from 146 patients with invasive breast cancer who received neoadjuvant chemotherapy. CD138^+^ PC infiltration was assessed by immunohistochemistry. Multiplexed fluorescent immunohistochemistry (mfIHC) with T and B cell markers was also conducted to elucidate the profile of immune cells.

**Results:**

Greater PC infiltration was observed in the pCR group (*p* = 0.028) and this trend was confirmed in another patient cohort. With mfIHC, we observed significantly more CD8^+^, T-bet^+^CD4^+^, and CD8^+^FOXP3^+^ T cells, total B cells and PCs in pCR cases. Such cases were also characterised by high expression of both PD-1 and PD-L1 on B cells and PCs. In patients with hormone receptor-negative tumours, high PC infiltration was correlated with significantly longer disease-free survival (*p* = 0.034).

**Conclusions:**

We found that higher PC infiltration in biopsy specimens before neoadjuvant chemotherapy was associated with pCR. With mfIHC, we also revealed that the local cytotoxic immune response was clearly enhanced in pCR cases, as was the infiltration of B cells including PCs. Moreover, higher PC levels were correlated with favourable outcomes in hormone receptor-negative breast cancer patients.

**Supplementary Information:**

The online version contains supplementary material available at 10.1186/s13058-021-01477-w.

## Background

Neoadjuvant chemotherapy (NAC) is offered to patients with relatively advanced breast cancer. NAC can identify patients responding well to chemotherapy and breast-conserving surgery might be an eligible option for those who achieved a significant response to NAC. Treatment effects assessed pathologically in surgical specimens may give useful information for subsequent adjuvant therapies. Meanwhile, the indications for NAC have become increasingly limited, based on treatment responsiveness [1]. Currently, more personalised treatments are being sought with molecularly targeted drugs in clinical trials. For instance, dual human epidermal growth factor receptor 2 (HER2) blockade, trastuzumab, and pertuzumab improved pathological complete response (pCR) rates for HER2-positive tumours [2, 3]. The efficacy of olaparib (PARP-inhibitor) in combination with paclitaxel in patients with *BRCA* mutations and/or a high homologous recombination deficiency score is being examined in a randomised trial (GeparOLA) [4]. Meanwhile, several studies have tried to find predictive markers for cytotoxic chemotherapies. The most crucial established marker is the intrinsic subtype, which is mostly determined by multi-gene profiling but for more practical use, this can be substituted by immunohistochemistry (IHC) in combination with measurements of oestrogen receptor (ER), progesterone receptor (PgR), and HER2. Luminal tumours generally respond poorly to chemotherapies [5, 6]. A major predictive marker of NAC success is Ki67, a nuclear protein associated with cellular proliferation [7, 8].

Tumour-infiltrating lymphocytes (TILs) are a prognostic marker for hormone receptor (HR)-negative breast cancer and patients with tumours with high TIL infiltration tend to have better outcomes [9–11]. TILs are now a direct treatment target as immune checkpoint inhibitors (ICIs) have been introduced for triple-negative (TN) breast cancer with PD-L1-positive TILs [12] and the amount of TILs is considered a predictive factor for ICI treatments [13]. Furthermore, TILs also have the potential to predict response to chemotherapy. High infiltration of TILs was reportedly related to higher pCR rates in TN and HER2-positive breast cancers [14]. Moreover, Seo et al*.* reported that pCR was frequently observed in tumours with high CD8^+^ T cell infiltration [15]. TILs are considered to play crucial roles in local cancer immunity. In HER2-positive tumours, an improved survival in patients with TIL-high tumours suggests that an immune response boosts the efficacy of trastuzumab [16, 17], via so-called antibody-dependent cell-mediated cytotoxicity. Galluzzi et al*.* suggested immunogenic tumour death induced by TILs in immunogenic chemotherapy such as anthracycline-based treatments [18]. However, how TILs boost the effect of chemotherapy is still largely unknown.

In daily pathological diagnoses, we noticed a high infiltration of plasma cells (PCs) in biopsy specimens from patients who achieved pCR after NAC. Local infiltration of PCs in breast cancer has generally been poorly investigated, but a few studies have reported a relationship with patient outcomes [19, 20]. Gentles et al*.* analysed tumour gene expression profiles and overall survival (OS) data from nearly 18,000 patients within a meta-analytical framework across 39 malignancies and found that PCs in primary tumours were significant predictors of favourable survival of patients with breast cancer and lung cancer [19]. A recent study demonstrated that patients with TN breast cancers with high densities of CD38^+^ PCs had significantly longer disease-free survival (DFS) [20]. Given the general role of PCs in synthesising and secreting immunoglobulins, how PCs contribute to anti-tumour immune responses at the primary site of breast cancer is largely unknown.

Considering this background, we hypothesised that PC infiltrated more in tumours that responded well to chemotherapy. To test this hypothesis in this study, we immunohistochemically investigated biopsy samples from patients who received NAC. As a result, high CD138^+^ PC infiltration was indeed frequently observed in patients who achieved pCR. Therefore, to ascertain the roles of PCs in the local immune microenvironment, we employed the multiplexed fluorescent method examining some profiles of T and B lymphocytes as well as PCs. Associations of PC infiltration and patient outcomes were also tested.

## Methods

### Patients

This study included 148 patients with invasive breast cancer who received NAC and underwent curative surgery at Juntendo University Nerima Hospital between 2006 and 2010. Of these, we retrospectively examined 146 patients whose clinical records and tumour samples were available. Clinicopathological features of the 146 patients are shown in Additional File 1. Mean age was 54.1 years. Subtype distributed as follows: luminal HER2-negative 53%, luminal HER2-positive 9%, HER2 type 19%, and TN 19%. NAC regimens: 132 (90.4%) patients received four cycles of CEF (C: cyclophosphamide 500 mg/m^2^, E: epirubicin 75 or 100 mg/m^2^, F: fluorouracil [5-FU] 500 mg/m^2^), followed by taxane (12 weeks of paclitaxel: 80 mg/m^2^ or four cycles of docetaxel: 75 mg/m^2^) prior to surgery; five (3.4%) patients received only CEF; and nine (6.2%) patients were given only taxane, due to conditions such as cardiac dysfunction. For patients with human epidermal growth factor receptor 2 (HER2)-positive tumours, trastuzumab was also administered simultaneously with taxane. Following surgery, HR-positive patients received adjuvant endocrine treatment. To test the association of PCs with pCR in HR-negative cases, another cohort of HR-negative tumours (*n* = 71), treated at Juntendo University Hospital, was also employed (see details in the Results section). This study was carried out with approvals from the ethics committee of Juntendo University Nerima Hospital (no.2020035) and Juntendo University Hospital (no.19–182). Patients could see the research plan on the website of the hospitals and were offered the choice to opt out of the study at any time.

### Pathological assessment and IHC

Pathological examinations were carried out by two pathologists at our hospital. Tumour grade was judged based on the Nottingham Histologic Score system and a grade III tumour was defined as high grade. Chemotherapy effects were determined employing surgical specimens, based on the General Rules for Clinical and Pathological Recording of Breast Cancer (the 18th edition published by the Japanese Breast Cancer Society) [21]. Briefly, grade 0 (no effect): no histological findings of treatment effect are observed, grade 1 (slightly effective): treatment changes in less than two-thirds of the invasive cancer tissue are seen, grade 2 (markedly effective): treatment changes in more than two-thirds of the invasive cancer tissue are seen, grade 3 (pCR): all invasive nests disappeared. In the current study, we defined pCR based only on the primary breast tumour, that is, without lymph node evaluation.

TIL amounts were determined using haematoxylin and eosin-stained tumour biopsy specimens, based on recommendations made by the International TILs Working Group [22]. Briefly, TILs in the stromal compartment (% stromal TILs), using the area of stromal tissue as a denominator, were determined semi-quantitatively. TILs were examined within the borders of the invasive tumour, and average TIL numbers in the tumour area, not focusing on hotspots, were assessed.

IHC was performed on biopsy specimens before NAC. ER and PgR were assessed based on the Allred scoring system [23]. Because the global cut-off value for HR at that time was 10%, HR-positive tumours at our institution were defined as having a total score of 4 or more. Considering that our patients received systemic treatments based on these criteria and recent studies suggest that tumours with less than 10% positivity respond poorly to endocrine therapies as they have a different molecular phenotype [24, 25], we retained our rules in the current study. Therefore, a tumour with less than 10% staining of cancer cell nuclei was considered HR-negative. HER2 was considered positive if the entire cell membrane of more than 10% of tumour cells showed strong staining, or *HER2/neu* gene amplification was confirmed by fluorescence in situ hybridisation. We used mouse monoclonal anti-Ki67 antibody, clone MIB-1 (Dako, Tokyo, Japan). The Ki67 labelling index was calculated for each biopsy specimen from a hotspot within a high-powered field (× 400). To achieve higher reproducibility, we counted cells with e-Count software (e-path, Kanagawa, Japan), developed for assessing nuclear IHC staining. Briefly, this software automatically counts the total number of positive and negative cells in the field and calculates the positive rate. For CD8 and PC marker CD138, clone C8/144B (Dako) and MI15 (Dako) were used, respectively. Positive immune cells in stromal areas were counted manually within a hotspot in a high-powered field (× 400), since the aforementioned software works only for nuclear staining. The researchers conducting these assessments were blinded to the effect of chemotherapy on the patients.

### Multiplexed fluorescent IHC and image analysis

For immune cell profiling we conducted multiplexed fluorescent immunohistochemistry (mfIHC). We used tyramide signal amplification with an Opal IHC kit (PerkinElmer, Waltham, MA, USA) according to the manufacturer's instructions. The paraffin-embedded block was cut into 4 μm sections. Primary antibodies used were: CD3 (clone SP7, Abcam, Tokyo, Japan), CD4 (clone 4B12, Leica Microsystems, Tokyo, Japan), CD8 (clone 4B11, Leica Microsystems), FoxP3 (D6O8R, Cell Signaling Technology, Danvers, MA, USA), T-bet (clone 4B10, Santa Cruz Biotechnology, Dallas, CA, USA), cytokeratin (clone AE1/AE3, Dako), CD20 (clone L26, Thermo Fisher Scientific, Tokyo, Japan), CD79a (clone JCB117, Dako) CD38 (clone SPC32, Leica Microsystems), PD-1 (clone EH33, Cell Signaling Technology), and PD-L1 (clone E1L3N, Cell Signaling Technology). We constructed two sets of panels, a T cell and a B cell panel. The former comprised CD3, CD4, CD8, Foxp3, and T-bet, while the latter comprised CD20, CD79a, CD38, PD-1, and PD-L1. Both panels also included cytokeratin to differentiate tumour and stromal areas, and DAPI for staining nuclei. For mfIHC, we employed CD38 and a combination of CD20 and CD79a for PC, i.e., PCs were defined as CD79a^+^CD20^−^CD38^+^ cells, since CD138 can be detected on immune cells as well as some cancer cells.

For each case, a whole slide was scanned at × 100 with an automated imaging system (Vectra ver. 3.0, PerkinElmer). They were exposed to the five filters (DAPI, FITC, CY3, TEXAS RED, and CY5) to ensure that each slide was in focus. Phenochart was used to annotate the tumour and stromal fields and whole specimens were captured with an average of 20 areas at × 200 magnification (sized 669 × 500 μm each). An image analysing software program (InForm, PerkinElmer) was used to segment cancer tissue into cancer cell nests (intra-tumoural) and the framework (stromal) region and to detect immune cells with specific phenotypes. Following the manufacturer’s instructions, manual training sessions for tissue segmentation and phenotype recognition using representative images of mfIHC were conducted. Then, automatic machine learning was repeated until the algorithm reached the necessary level of confidence before performing the final evaluation. Representative images of tissue segmentation and cell phenotype recognition are shown in Additional File 2. Infiltrating immune cells were quantified using an analytic software program (Spotfire, TIBCO, Palo Alto, CA, USA) and then calculated per area.

### Statistical analysis

Statistical analyses were performed using JMP 14.2 statistical software (SAS Institute, Inc., Cary, NC, USA). A logistic regression model was constructed to identify factors characterising pCR cases. For the full-model analysis, we first selected variables according to their clinical significance: age, tumour grade, ER and HER2 statuses, and TIL. Comparisons of mean values for PCs were performed on unpaired data using the Welch's t-test. A Cox proportional hazard model was employed for predicting patient outcomes. This included pathological invasive size of remnant disease and lymph node involvement, along with the five aforementioned factors. Kaplan–Meier curves were estimated and the log-rank test was applied for comparisons of survival distributions between the two patient groups. A *p* < 0.05 was considered statistically significant.

## Results

### Clinicopathological features associated with pCR

The overall pCR rate was 17% (25 cases). High grade, high Ki67 labelling index, PgR-negative and HER2-positive tumours showed a significantly higher pCR rate (Table 1; *p* = 0.017, *p* < 0.001, *p* = 0.033, and *p* = 0.001, respectively). Tumours with a high infiltration of TIL and CD138 also had a higher frequency of pCR (*p* = 0.002 and *p* = 0.039, respectively). On multivariate analysis, HER2 status and TIL remained as independent factors associated with pCR (*p* = 0.002 and *p* = 0.003, respectively).

**Table 1:** Clinicopathological features and pCR

Variables	pCR		Non-pCR		Univariate			Multivariate		
					OR	95%CI	*p* value	OR	95%CI	*p* value
*n*	25		121							
Age (mean)	55.2		53.9		1.70*	0.22–13.31	0.611	2.30*	0.16–33.38	0.534
Histology										
NST	24	(96%)	107	(88%)	3.14	0.39–25.05	0.28			
Others	1	(4%)	14	(12%)						
Tumour grade										
High	15	(60%)	41	(34%)	2.93	1.21–7.09	0.017	2.43	0.83–7.40	0.107
Low	10	(40%)	80	(66%)						
Ki67 (%, mean)	29.0		16.3		214.58*	25.26–2583.97	< 0.001			
ER										
Positive	11	(44%)	78	(64%)	0.43	0.18–1.04	0.06	0.54	0.15–1.79	0.314
Negative	14	(56%)	43	(36%)						
PgR										
Positive	7	(28%)	63	(52%)	0.36	0.14–0.92	0.033			
Negative	18	(72%)	58	(48%)						
HER2										
Positive	14	(56%)	27	(22%)	4.43	1.80–10.88	0.001	5.22	1.86–15.53	0.002
Negative	11	(44%)	94	(78%)						
TIL (%, mean)	42.0		22.8		8.49*	2.27–32.70	0.002	9.66*	2.17–46.07	0.003
CD8 (mean)	165.1		156.7		1.47*	0.10–16.48	0.764			
CD138 (mean)	100.9		61.3		7.00*	1.11–43.56	0.039			

*ER* oestrogen receptor, *PgR* progesterone receptor, *HER2* human epidermal growth factor receptor 2, *TIL* tumour-infiltrating lymphocytes, *NST* no special type, *OR* odds ratio, CI confidence interval, *TIL* tumour-infiltrating lymphocytes, *NST* no special type, *OR* odds ratio, *CI* confidence interval

^*^Range of the odds ratio

### Infiltrations of plasma cells and other immune cells according to chemo-effect

**Fig. 1:**
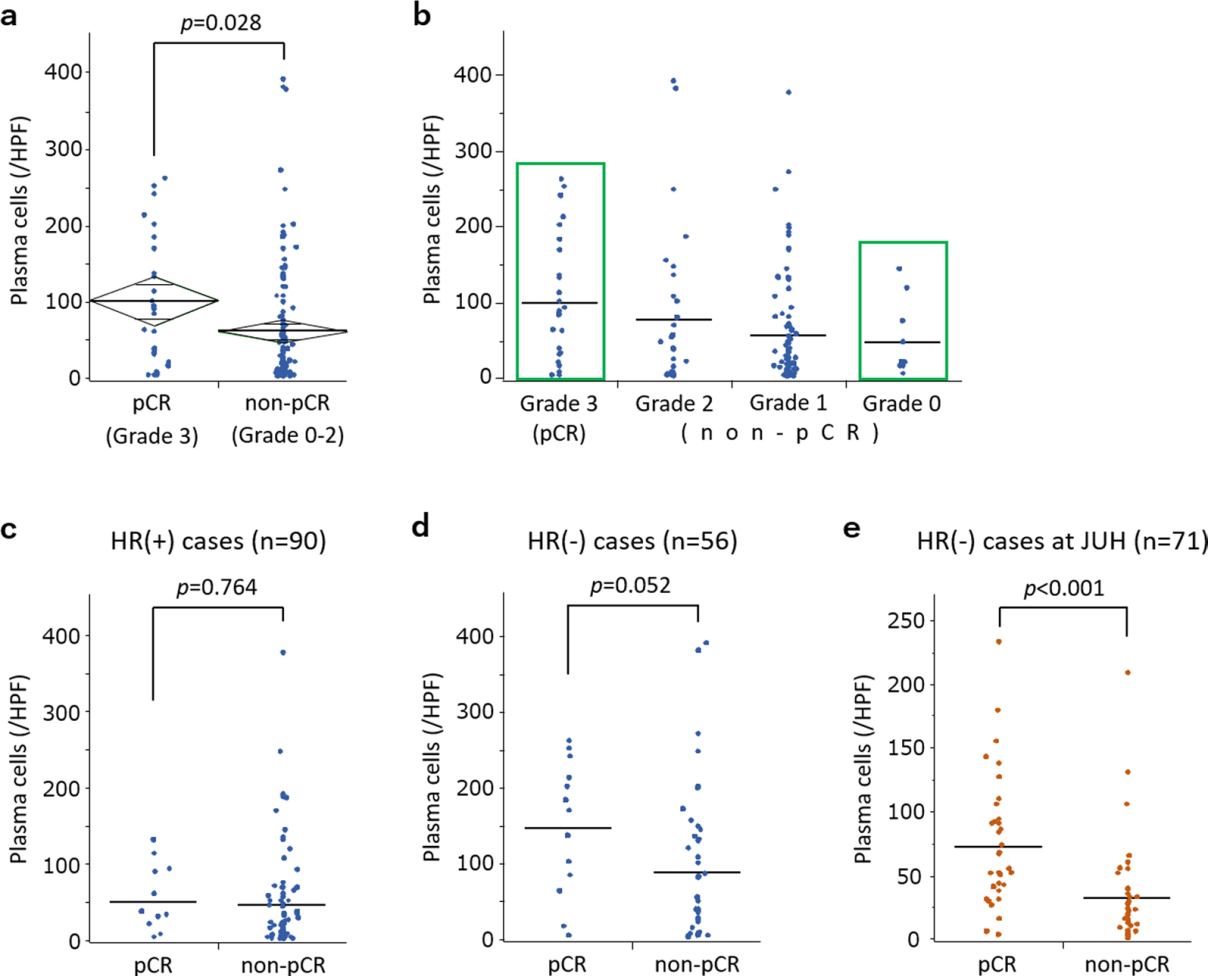
Plasma cell infiltration according to chemo-effect. **a**, **b** Comparisons of PC infiltration according to chemo-effect (pCR; *n* = 25, non-pCR; *n* = 121). Horizontal bars indicate mean values. Green rectangles indicate cases in which samples were further investigated with mfIHC. **c**, **d** PC infiltration according to chemo-effect and HR status. c HR-positive patients (*n* = 90) and d HR-negative patients (*n* = 56). **e** Comparisons of PC infiltration according to chemo-effect in another cohort of HR-negative patients (*n* = 71) from Juntendo University Hospital (JUH)

To focus on the relationship between PC infiltration and chemo-effect, we further examined the distributions of PCs in relation to chemo-effect. Comparing mean values of PC, we observed significantly more PC infiltration in the pCR group (Fig. 1a, *p* = 0.028). When non-pCR cases were further classified based on chemo-effect, we found PC number was dependent on chemo-effect, where mean values decreased from Grade 3 to 0 (101, 78, 57, and 48, respectively; Fig. 1b). When PC infiltrations were assessed according to HR status, no differences were observed in HR-positive tumours (*p* = 0.764; Fig. 1c), whereas a trend of correlation between PC infiltration and chemo-effect was observed in HR-negative cases (*p* = 0.052; Fig. 1d). To test whether this correlation would be observed in another cohort, we used HR-negative tumours (*n* = 71) from Juntendo University Hospital (JUH). Clinicopathological features of these patients are shown in Additional File 3. In this cohort, the correlation between PC infiltration and chemo-effect was confirmed (*p* < 0.001), as more PC infiltrations were observed in pCR cases (Fig. 1e).

**Fig. 2:**
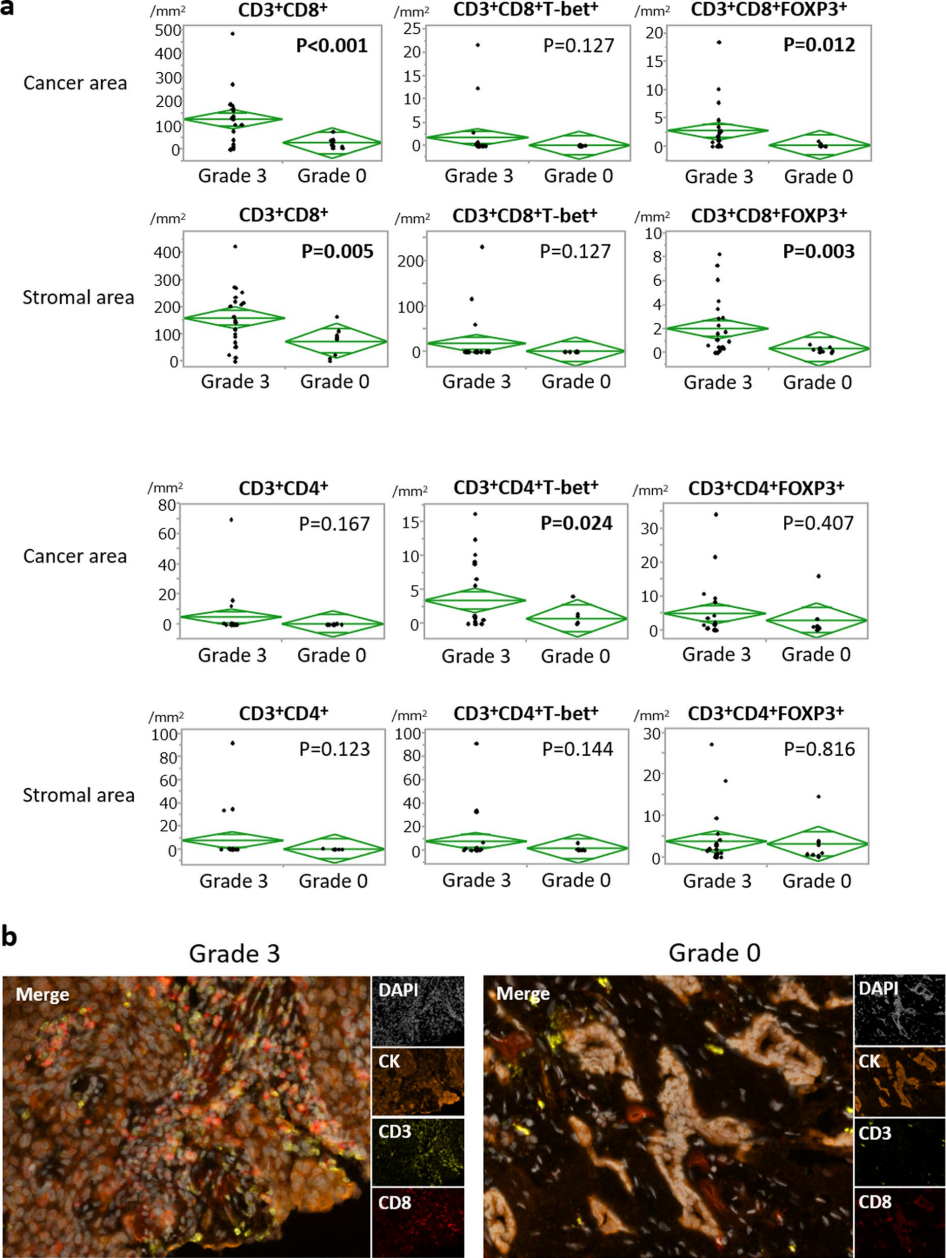
Comparison of immune cell profiling on T cell panel according to chemo-effect. **a** Comparisons of immune cells on T cell panel according to chemo-effect. We compared grade 3, i.e., pCR (*n* = 25), and grade 0 (*n* = 10) tumours employing mfIHC. Assessments in cancer and stromal areas are separately indicated. **b** Representative images of CD3^+^CD8^+^ T cell infiltrations by mfIHC. Cytokeratin (orange), CD3 (yellow), and CD8 (red)

**Fig. 3:**
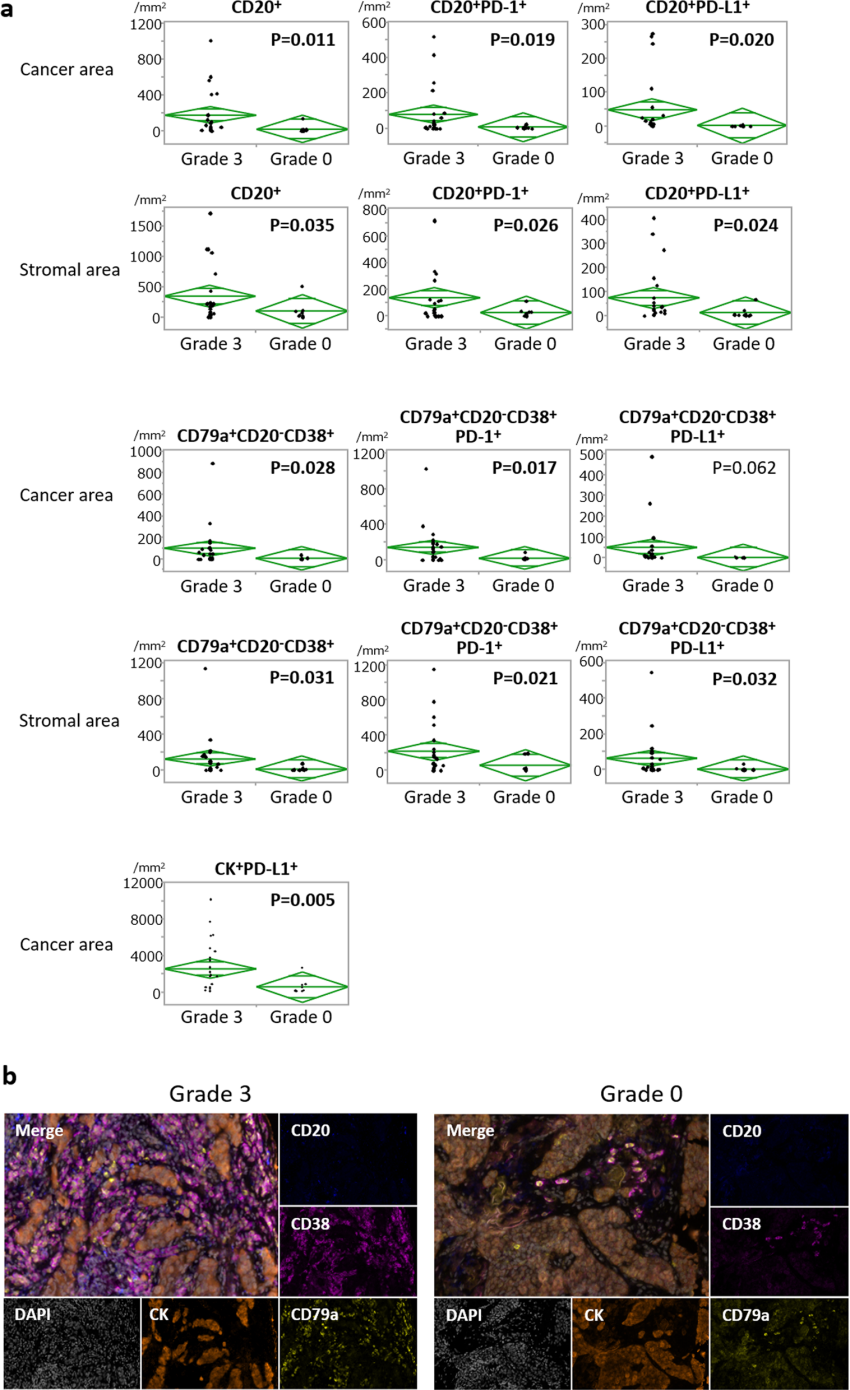
Comparison of immune cell profiling on B cell panel according to chemo-effect. **a** Comparisons of immune cells on B cell panel according to chemo-effect. Expression of PD-L1 in cancer cells is also shown. **b** Representative images of PC infiltrations by mfIHC. Cytokeratin (orange), CD20 (blue), CD38 (magenta), and CD79a (yellow)

To elucidate roles of PCs, we selected cases with Grade 3 (pCR; *n* = 25) and Grade 0 (*n* = 10) for further examination with mfIHC (indicated by green rectangles in Fig. 1b). On the T cell panel, we observed significantly more CD3^+^CD8^+^ T-lymphocytes in pCR cases, both in cancer and stromal areas (Fig. 2a) (*p* < 0.001 and *p* = 0.005, respectively). Representative images of CD3^+^CD8^+^ T cell infiltrations are shown in Fig. 2b. CD3^+^CD4^+^T-bet^+^ T-lymphocytes were also more frequently observed in the cancer area of pCR cases (*p* = 0.024), as well as CD3^+^CD8^+^FOXP3^+^ T cells (*p* = 0.012). On the B cell panel, significantly more total B-lymphocytes and PCs were observed in pCR cases, both in cancer and stromal areas (Fig. 3a). Representative images of PC infiltrations by mfIHC are shown in Fig. 3b. Notably, tumours of the pCR group frequently had more infiltration of both PD-1^+^ and PD-L1^+^ B cells and PCs. Significantly higher expression of PD-L1 in cancer cells was also observed in the pCR group (*p* = 0.005).

### Patient outcomes and PC infiltration

**Fig. 4:**
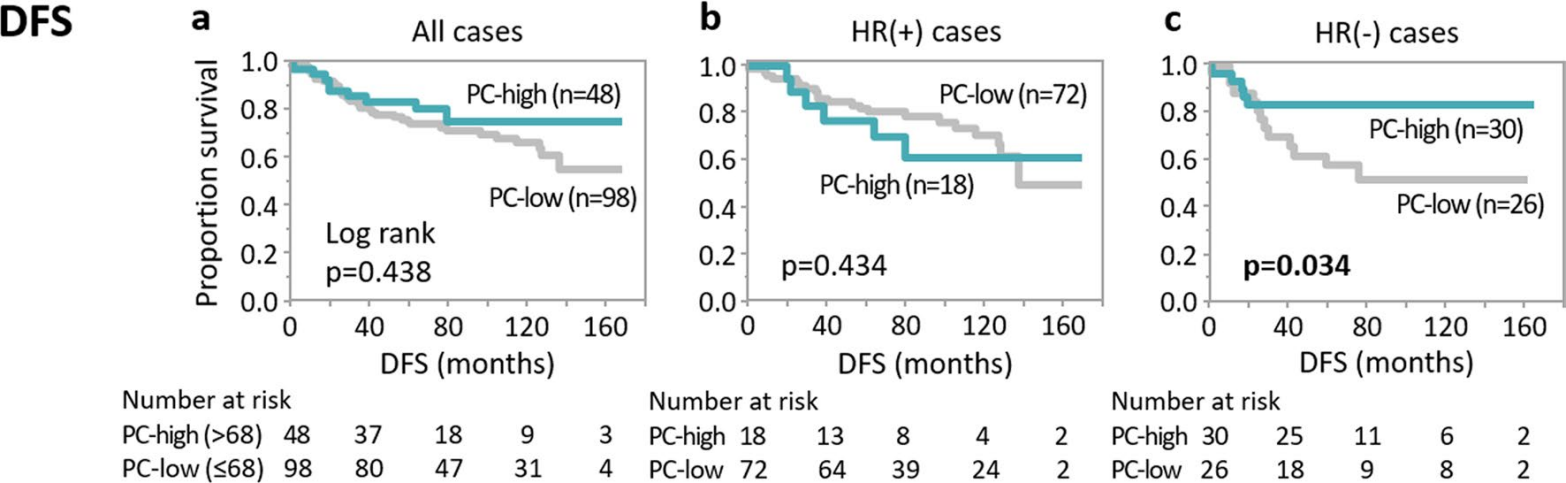
Kaplan–Meier curves of disease-free survival according to plasma cell infiltration. Disease-free survival according to PC infiltration in **a** all participants (*n* = 146), **b** HR-positive patients (*n* = 90), and **c** HR-negative patients (*n* = 56). Light green lines indicate the PC-high group, while grey lines represent the PC-low group

During the median 78-month observation period (range: 14–168) for the 146 patients, 44 (30%) developed distant metastases and 34 (23%) died due to breast cancer. To examine the relationships between clinicopathological factors and patient outcomes, a Cox proportional hazard model was constructed. As shown in Table 2, remnant tumour size and lymph node metastasis were independent factors associated with shorter DFS (*p* = 0.048 and *p* = 0.007, respectively). The hazard ratio of having high PC infiltration was 0.36, indicating that patients with high PC tumours might survive longer. Therefore, we further focused on the relationship between PC expression and DFS and drew Kaplan–Meier curves. The mean value of PC numbers of 64 was employed as cut-off to distinguish PC-high and -low groups. Interestingly, Kaplan–Meier curves show that patients with HR-negative tumours with high PC infiltration had significantly longer DFS (*p* = 0.034), a trend not observed in patients with HR-positive tumours (Fig. 4). No statistically significant differences were identified concerning OS (Additional File 4). For reference, Kapan–Meier curves of patient outcomes according to TIL are also shown in Additional File 5.

**Table 2:** Clinicopathological features and disease-free survival

Variables	Univariate			Multivariate		
	HR	95%CI	*p* value	HR	95%CI	*p*-value
Age	0.67*	0.15–2.89	0.590	0.52*	0.10–2.62	0.426
Histology						
NST vs others	0.57	0.25–1.28	0.172			
Residual tumour size in breast	5.30*	1.68–14.44	0.006	4.11*	1.01–14.86	0.048
Residual tumour in lymph node						
Positive vs negative	2.98	1.62–5.49	< 0.001	2.54	1.29–5.09	0.007
Tumour grade						
High vs low	1.27	0.69–2.31	0.444	1.33	0.63–2.75	0.443
Ki67	3.22*	0.91–10.67	0.070			
ER						
Positive vs negative	0.91	0.49–1.67	0.758	0.66	0.26–1.62	0.360
PgR						
Positive vs negative	1.04	0.57–1.88	0.905			
HER2						
Positive vs negative	0.93	0.48–1.82	0.838	1.04	0.45–2.34	0.920
TIL	0.96*	0.32–2.50	0.938	1.22*	0.38–3.52	0.730
CD8	1.81*	0.30–8.60	0.497			
CD138	0.36*	0.06–1.63	0.201			

*ER* oestrogen receptor, *PgR* progesterone receptor, *HER2* human epidermal growth factor receptor 2, *TIL* tumour-infiltrating lymphocytes, *NST* no special type, *HR* hazard ratio, *CI* confidence interval

^*^Range of the hazard ratio

## Discussion

To the best of our knowledge, this is the first report of greater PC infiltration in primary tumours in pCR cases. The major functions of PC are antigen presentation and humoral immunity by antibody production. While the roles of PCs in local tumour immunity are largely unknown, tumour-specific IgG1 antibodies produced by PCs are known to have anti-tumour effects through antibody-dependent cell cytotoxicity (ADCC) [26]. Moreover, there is some evidence that PC can contribute to cytotoxic immune responses through the release of cytokines, such as IL-12 and IFN-γ [26, 27]. For instance, IL-12 induces dendritic cell migration and drives CD8^+^ T cell activity [28, 29]. There is also a possibility that the large quantity of non-specific antibodies produced by PCs may boost other immune cells with the Fc region of IgG, as many cells express Fc receptors [30–32]. We believe that the increased expression of PD-L1 on PCs observed in our study is also evidence of the direct interaction between PCs and other immune cells.

With mfIHC, we revealed that the cytotoxic immune responses were clearly enhanced in local tumours, as significantly more CD8^+^ T cells and T-bet^+^CD4^+^ T cells were observed in pCR cases. Meanwhile, almost all B cell lineage cells (CD79a^+^ B cells) infiltrated in pCR cases, positively correlating with a state of enhanced cell-mediated immunity. For instance, IL-12, produced from B cell lineage cells, promotes the proliferation of T and NK cells and induces cytotoxicity [33, 34]. Thus, our observations are plausible. Interestingly, PCs in such tumours highly expressed PD-L1, indicating that these cells also have an inhibitory effect. PD-L1 itself promotes the differentiation of naive T cells into regulatory T cells [35]. Moreover, PD-L1^+^ PCs have been reported to suppress helper T cells (including T-bet^+^CD4^+^ T cells) and B cells by producing IL-10 [36]. Our data therefore suggest that PCs are involved in the regulation of tumour immunity.

In our study, the amount of TIL was strongly associated with pCR, but not with patient outcomes. In contrast, PCs were associated with a favourable prognosis in HR-negative patients and patients with a high PC infiltration in their tumours before NAC had significantly longer DFS. This trend, however, was not observed in patients with HR-positive breast cancer. Our results are consistent with previous reports suggesting PCs as a good prognostic factor in TN breast cancer [20, 37]. In the study by Yeong et al*.*, a higher expression level of IgG genes, probably reflecting PC functions, were also correlated with a better clinical outcome [20]. Assessment with specific immune cell markers may have a stronger prognostic potential than TILs in general, since TILs contain a variety of immune cells. PC has shown their potential as such a marker in the current study, as CD8-positive T cells are known to be such a prognostic marker for TNBC. Concerning how PCs contribute to improving patient outcomes, Gentles et al*.* suggested that antigen presentation by PCs may be crucial for the antigen-driven processes, which eventually induce B cell clonal expansion and emergent humoral immune responses [19]. Based on our data, we speculate that the presence of tumour-infiltrating PCs in pre-treatment specimens reflects an adaptive immune response to the cancer by the host immune system.

A major limitation of the current study is a lack of functional analysis of immunocompetent cells. To clarify the effect of a complexed immune network on the therapeutic effect, it is necessary to perform genome-wide analysis on more cases. Moreover, the lifespan of PCs in vivo is very short, in the range of several weeks, and more detailed analyses are needed to elucidate the role of PCs in local tumour lesions. As for CD138 assessment in IHC, some issues still remain to be solved. Intra- and inter-examiner reproducibility as well as the significance of intra-tumoural heterogeneity of CD138 expression should be tested in other studies. Moreover, determining the cut-off value of CD138 for distinguishing pCR and non-pCR cases might merit further studies with larger sample sizes.

## Conclusions

In summary, we found that higher PC infiltration in pre-treatment tumour biopsy specimens was associated with pCR after NAC. mfIHC revealed an enhanced local cytotoxic immune response and high infiltration of B cells including PCs in pCR cases. Moreover, PCs were associated with a better prognosis in HR-negative breast cancer patients.

## Supplementary Information

**Additional file 1**. Clinicopathological features of the 146 patients.

**Additional file 2**. Representative images of tissue segmentation and cell phenotype recognition. **a** Employing an image analysing software program, tissue segmentation was conducted. Tumour area, stromal area, and other area were recognised with red, green, and blue, respectively. **b** Cell phenotyping was also conducted with markers specific to each cell; cancer cells (orange), stromal cells (green), CD4^+^ T cells (blue), and CD8^+^ T cells (red). A combination of the tissue segmentation and cell phenotyping enabled separate assessments of infiltrating immune cells in intra-tumoural and stromal regions.

**Additional file 3**. Clinicopathological features of hormone receptor-negative patients (*n* = 71) from JUH.

**Additional file 4**. Kaplan–Meier curves of overall survival according to plasma cell infiltration. Overall survival according to PC infiltration in **a** all participants (*n* = 146), **b** HR-positive patients (*n* = 90), and **c** HR-negative patients (*n* = 56). Light green curves denote patients with PC-high and grey curves PC-low tumours.

**Additional file 5**. Kaplan–Meier curves of patient outcomes according to TIL. Disease-free survival and overall survival according to TIL infiltration in **a** and **d** all participants (*n* = 146), **b** and **e** HR-positive patients (*n* = 90), and **c** and **f** HR-negative patients (*n* = 56). Blue curves denote TIL-high (> 26%) tumours and grey curves indicate patients with TIL-low (≤ 26%) tumours.

## Data Availability

Data sharing not applicable to this article as no datasets were generated.

## Abbreviations

ADCC: Antibody-dependent cell cytotoxicity
AUC: Area under the curve
DFS: Disease-free survival
ER: Oestrogen receptor
HR: Hormone receptor
HER2: Human epidermal growth factor receptor 2
IHC: Immunohistochemistry
mfIHC: Multiplexed fluorescent immunohistochemistry
NAC: Neoadjuvant chemotherapy
OS: Overall survival
PC: Plasma cell
pCR: Pathological complete response
PgR: Progesterone receptor
ROC: Receiver operating characteristic
TIL: Tumour-infiltrating lymphocyte
TN: Triple-negative
