# Supplementary figures and images for: Plasma cell infiltration and treatment effect in breast cancer patients treated with neoadjuvant chemotherapy
Source: Breast Cancer Res. 2021 Oct 29;23:99. doi: 10.1186/s13058-021-01477-w (PMC8555250; doi:10.1186/s13058-021-01477-w)

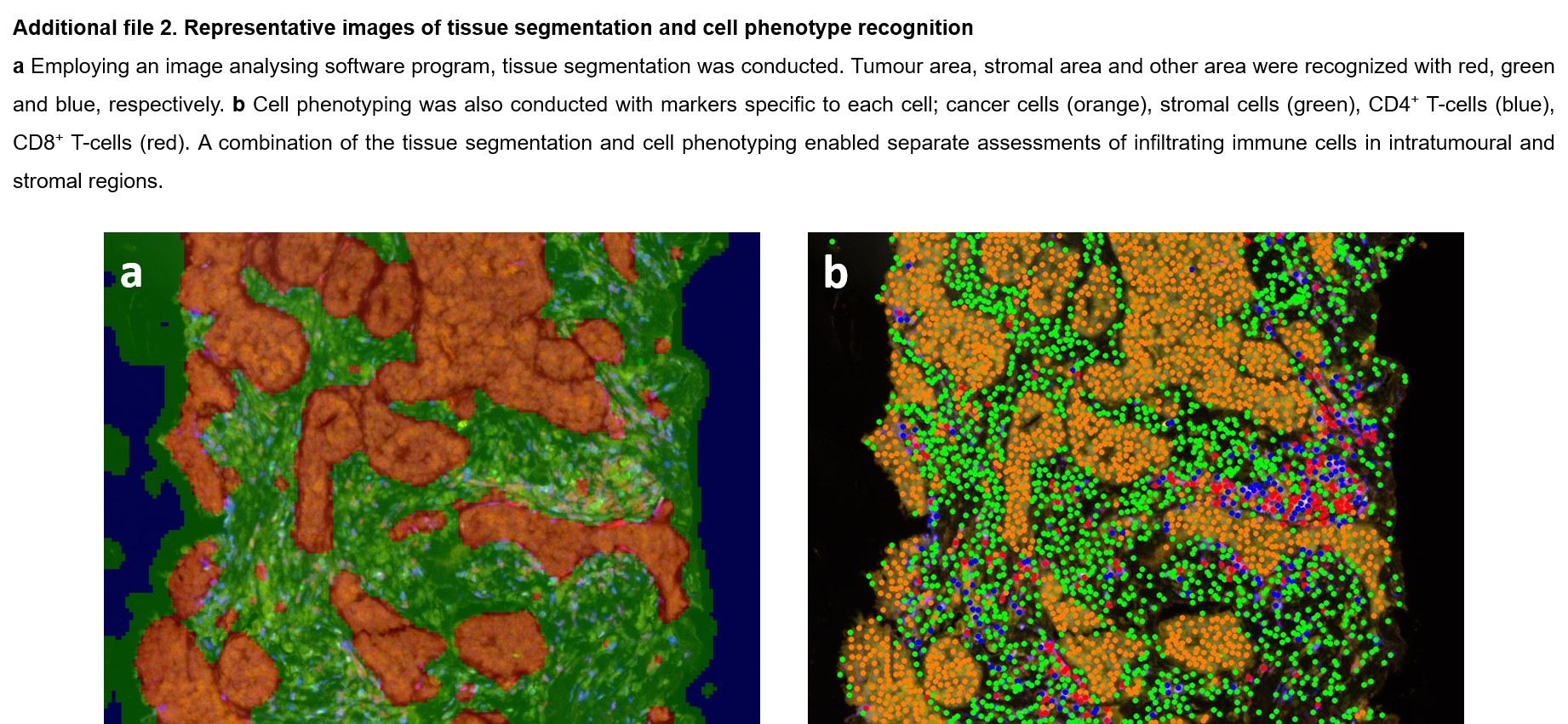

Supplement: Supplementary file 2 — Additional file 2. Representative images of tissue segmentation and cell phenotype recognition. a Employing an image analysing software program, tissue segmentation was conducted. Tumour area, stromal area, and other area were recognised with red, green, and blue, respectively. b Cell phenotyping was also conducted with markers specific to each cell; cancer cells (orange), stromal cells (green), CD4+ T cells (blue), and CD8+ T cells (red). A combination of the tissue segmentation and cell phenotyping enabled separate assessments of infiltrating immune cells in intra-tumoural and stromal regions. [file 13058_2021_1477_MOESM2_ESM.tif]

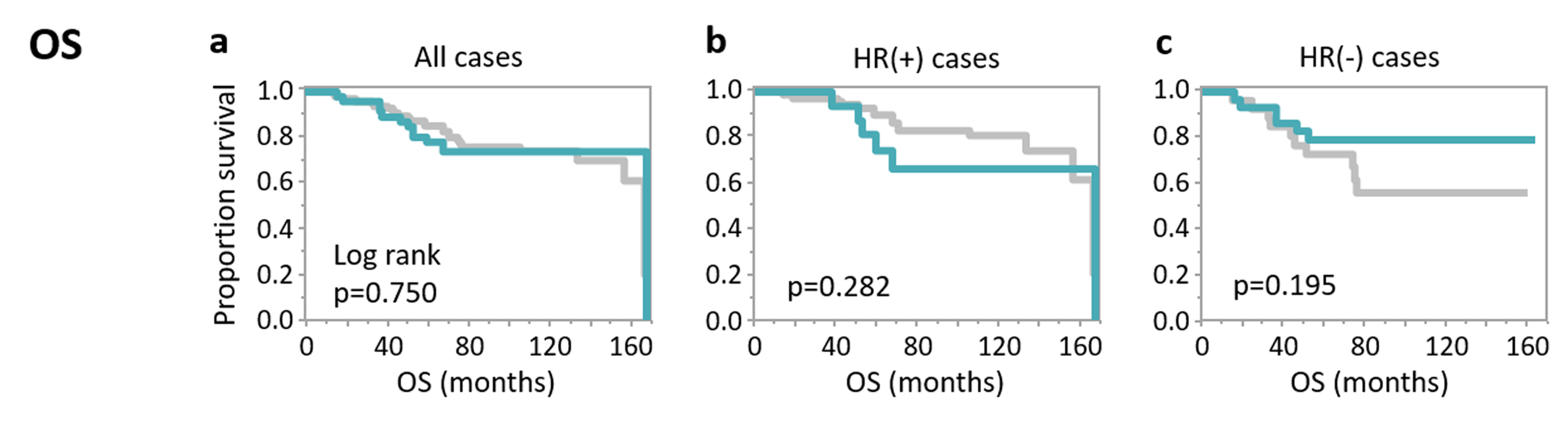

Supplement: Supplementary file 4 — Additional file 4. Kaplan–Meier curves of overall survival according to plasma cell infiltration. Overall survival according to PC infiltration in a all participants (n = 146), b HR-positive patients (n = 90), and c HR-negative patients (n = 56). Light green curves denote patients with PC-high and grey curves PC-low tumours. [file 13058_2021_1477_MOESM4_ESM.tif]

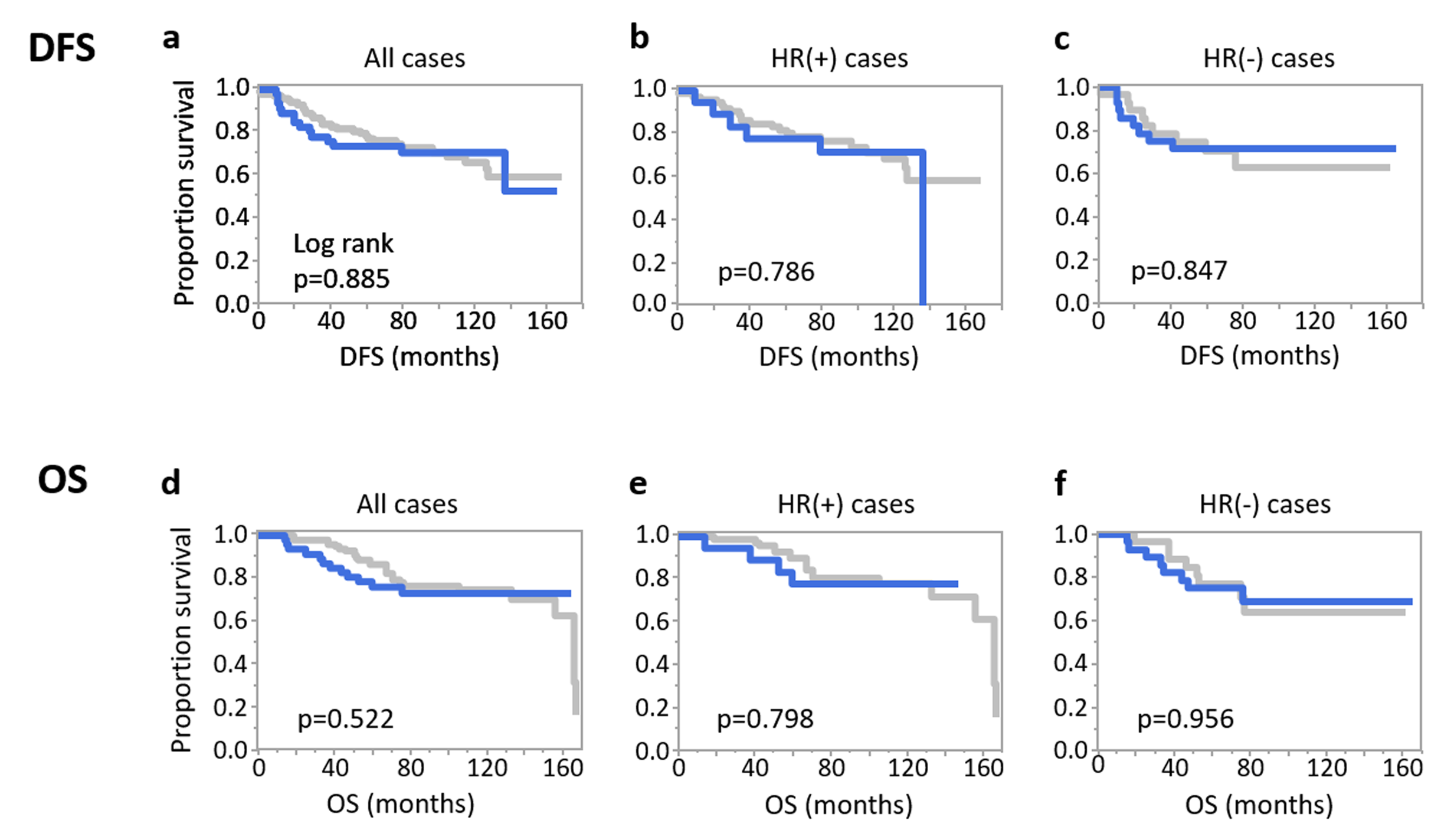

Supplement: Supplementary file 5 — Additional file 5. Kaplan–Meier curves of patient outcomes according to TIL. Disease-free survival and overall survival according to TIL infiltration in a and d all participants (n = 146), b and e HR-positive patients (n = 90), and c and f HR-negative patients (n = 56). Blue curves denote TIL-high (> 26%) tumours and grey curves indicate patients with TIL-low (≤ 26%) tumours. [file 13058_2021_1477_MOESM5_ESM.tif]
